# Short stop mediates axonal compartmentalization of mucin-type core 1 glycans

**DOI:** 10.1038/srep41455

**Published:** 2017-02-02

**Authors:** Takaaki Kinoshita, Chikara Sato, Takashi J. Fuwa, Shoko Nishihara

**Affiliations:** 1 Laboratory of Cell Biology, Department of Bioinformatics, Graduate School of Engineering, Soka University, 1-236 Tangi-machi, Hachioji-shi, Tokyo 192-8577, Japan; 2 Biomedical Research Institute, National Institute of Industrial Science and Technology (AIST) Tsukuba Central 6 and 2, 1-1-1 Higashi, Tsukuba-shi, Ibaraki 305-8566, Japan

## Abstract

T antigen, mucin-type core 1 *O*-glycan, is highly expressed in the embryonic central nervous system (CNS) and co-localizes with a *Drosophila* CNS marker, BP102 antigen. BP102 antigen and Derailed, an axon guidance receptor, are localized specifically in the proximal axon segment of isolated primary cultured neurons, and their mobility is restricted at the intra-axonal boundary by a diffusion barrier. However, the preferred trafficking mechanism remains unknown. In this study, the major *O*-glycan T antigen was found to localize within the proximal compartments of primary cultured *Drosophila* neurons, whereas the *N*-glycan HRP antigen was not. Ultrastructural analysis by atmospheric scanning electron microscopy revealed that microtubule bundles cross one another at the intra-axonal boundary, and that T antigens form circular pattern before the boundary. We then identified Short stop (Shot), a crosslinker protein between F-actin and microtubules, as a mediator for the proximal localization of T antigens; null mutation of *shot* cancelled preferential localization of T antigens. Moreover, F-actin binding domain of Shot was required for their proximal localization. Together, our results allow us to propose a novel trafficking pathway where Shot crosslinks F-actin and microtubules around the intra-axonal boundary, directing T antigen-carrying vesicles toward the proximal plasma membrane.

The single axons of primary cultured *Drosophila* neurons are considered to have proximal and distal compartments^1^. Derailed (DRL), an axon guidance receptor of *Drosophila*, and the BP102 antigen, a *Drosophila* central nervous system (CNS) marker, are localized mainly in the proximal region of axons, whereas ROBO3, a member of the axon guidance receptor roundabout (ROBO) family, is restricted in the distal region of axons. The proximal region, which is termed the proximal segment, is closest to the cell body (soma). A diffusion barrier restricts the mobility of membrane molecules at the compartment boundary in *Drosophila*^1^. In mammals, the single axons of spinal neurons are also compartmentalized^2^. Thus, intra-axonal compartmentalization seems to be evolutionarily conserved. The proximal localization of DRL is probably achieved by transporting molecules selectively to the plasma membrane in proximal axon segments, and retrieving incorrectly localized molecules from the distal segment by endocytosis^1^. Recently, we analysed the precise localization of *Drosophila* CNS marker BP102 antigen, F-actin, and microtubules near the boundary of the intra-axonal segment by atmospheric scanning electron microscopy (ASEM)^3^. ASEM allows *in situ* correlative light and electron microscopy of cells in aqueous solution, in an open atmospheric environment^4^,^5^. The images showed that BP102 antigens accumulate near the intra-axonal boundary, where they form a special polygonal frame-like structure^3^. F-actin is abundant on the proximal side of the boundary, and the tubulin bundles in the axon often cross each other at the boundary. However, the mechanism underlying the preferential trafficking of molecules to the proximal axon segment is largely unknown.

Previously, we reported that T antigen (Galβ1–3GalNAcα1-Ser/Thr) (Fig. 1a), mucin-type core 1 *O*-glycan, is highly expressed in the nervous system of *Drosophila* embryos^6^. T antigen is the most common structure of mucin-type *O*-glycans and is synthesized by T antigen synthetase (core 1 β1,3-galactosyltransferase1 (C1β3GalT1)), which is evolutionarily conserved^7^,^8^,^9^. In *Drosophila* and mammals, T antigen is expressed and functions in the nervous and blood systems^10^,^11^,^12^,^13^. Recently, we reported that T antigen is involved in the localization of neuromuscular junction (NMJ) boutons and in the synaptogenesis of NMJs^14^. Another group reported that loss of *Drosophila C1*β*3GalT1* causes elongation of the ventral nerve cord and malformation of hemispheres in the brain^15^. The results indicate that T antigen plays a significant role in the formation of neural networks.

The protein spectraplakin, which is called Short stop (Shot) in *Drosophila*, forms cross-links between F-actin and tubulin^16^. In *Drosophila*, Shot is required for the extension of axons to target muscles^17^, midline axon repulsion^18^, and microtubule organization during neuronal growth^19^. However, it is still unclear whether Shot is involved in axonal trafficking.

In this study, we found that T antigen, mucin-type core 1 *O*-glycan, is localized specifically in the proximal axon segment of primary cultured neurons, whereas the *Drosophila* neuronal marker horse-radish peroxidase (HRP) antigen^20^,^21^, a type of *N*-glycan, is localized in the whole axon fibre. T antigens accumulate near the intra-axonal boundary in a circular pattern. Moreover, we identified Shot as a segmentation factor for the proximal localization of T antigen. Finally, we found that the F-actin binding domain of Shot is required for this preferential localization.

## Results

### T antigen localization in the proximal axon segment

**Figure 1:**
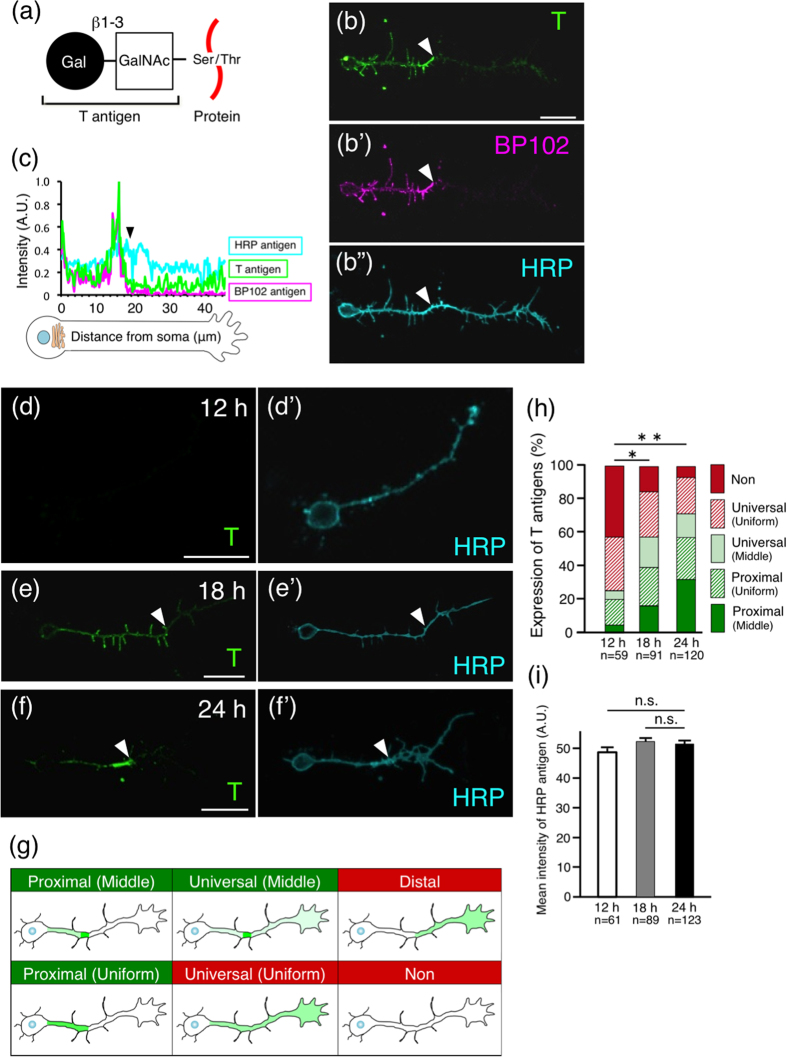
T antigen localization in the proximal axon segment. Filled arrowheads indicate the intra-axonal boundary. (**a**) Diagram depicting T antigen. Gal: galactose. GalNAc: *N*-acetylgalactosamine. (**b**) Localization of T, BP102, and HRP antigens on the surface of wild-type 24-h-cultured primary neurons. T and BP102 antigens localized on the surface of proximal axon segments and HRP antigens on the whole axon. (**c**) Fluorescence intensities of T, BP102, and HRP antigens along axons. (**d**–**f’**) Localization of T and HRP antigens on the surface of neurons cultured for 12 h (**d**,**d’**), 18 h (**e**,**e’**), and 24 h (**f**,**f’**). (**g**) Diagram depicting the 6 categories used to classify label-localization in neurons (see Methods). Neurons with proximal localization of T antigens (green) were classified into three groups: Proximal (Middle), Proximal (Uniform), and Universal (Middle). In the other categories, T antigen was not confined to the proximal axon segments of the neurons (red). (**h**) Histogram showing the localization of T antigens on the surface of neurons cultured for 12 to 24 h. *P*-values were calculated using the G-test. **p = 1.6 × 10^−10^, *p = 8.9 × 10^−6^. With increased culture time, the percentage of neurons with proximal localization of surface T antigen increased (green). (**i**) The mean intensity of HRP antigens on the whole surface of the axons ± SD. *P*-values were calculated using Dunnett’s test. n.s. = 0.27 (between 12 h and 24 h), n.s. = 0.81 (between 18 h and 24 h). Scale bars: 10 μm (**b**,**d**–**f**).

We previously reported that T antigen (Fig. 1a), a major *O*-glycan in *Drosophila*, is expressed in the embryonic CNS and co-localized with BP102 antigen, a marker of the *Drosophila* CNS^6^ (Supplementary Fig. S1). As BP102 antigens localize within the proximal axon segment of primary cultured neurons of *Drosophila*^1^, we expected T antigens to also localize there.

When 24-h primary-cultured neurons were labelled with PNA lectin (recognizes T antigens) without permeabilization, we found that T antigens tended to localize on the surface of the proximal axon segment (Fig. 1b,c,h and Table 1). Because PNA signals disappeared in *C1*β*3GalT1* mutant neurons (Supplementary Fig. S2), PNA lectin recognizes T antigen specifically in *Drosophila* neurons. The intra-axonal boundaries defined by surface T antigens and BP102 antigens were at the same position (Fig. 1b,c; filled arrowheads). Another *Drosophila* neuronal marker HRP antigen^20^,^21^ (Supplementary Fig. S1), which is an *N*-glycan, was then found to be distributed on the surface of the whole axon (Fig. 1b”). To statistically analyse the localization of the labels, we classified the neurons into six groups describing the observed situation: Non, Universal (Uniform), Universal (Middle), Proximal (Uniform), Proximal (Middle), and Distal (see Methods) (Fig. 1g and Table 1). Neurons with proximally-specific T antigens were categorized as Proximal (Middle), Proximal (Uniform), or Universal (Middle), respectively (Fig. 1g and Table 1; green and bold, respectively), whereas the other neurons were categorized as Non, Universal (Uniform), or Distal (Fig. 1g and Table 1; red and plain, respectively). The number of neurons defined as distal was less than 5.5% for T antigen. Thus, distal labelling is recorded in Tables 1 and 2 but not illustrated in the figures. Because the percentage of neurons with proximal localization of surface T antigens (Fig. 1h and Table 1; green and bold, respectively) was much larger than the percentage displaying other localizations (Fig. 1h and Table 1; red and plain, respectively), we conclude that T antigens localize specifically to proximal axon segments in 24-h neuron cultures.

Next, we examined changes in the surface localization of T antigens on cultured neurons during neural development (Fig. 1d–h). In 12-h primary cultures, only 25% of the neurons displayed proximal T antigen (Fig. 1h; green), and for over 40% of the neurons, T antigen expression was below the detection limit (Non) (Fig. 1d,h). In 18- and 24-h primary cultures, the percentage of neurons displaying proximal T antigen localization increased to 57% and 70%, respectively (Fig. 1e–h). Furthermore 21-d primary-cultured neurons still showed proximal T antigen localization (Supplementary Fig. S3). This finding indicates that that axonal compartmentalization is maintained during long-term culture. In contrast, HRP antigens were always localized on the whole axonal membrane of neurons (Fig. 1d’,e’,f’) and were expressed at similar levels in 12- to 24-h primary cultures (Fig. 1i). These results show that T antigens are gradually localized to the surface of proximal axon segments during neurogenesis, whereas HRP antigens are not. Therefore, we analysed the neurons from 24-h primary cultures in the following experiments.

### Ultrastructural features characteristic to the intra-axonal boundary

**Figure 2:**
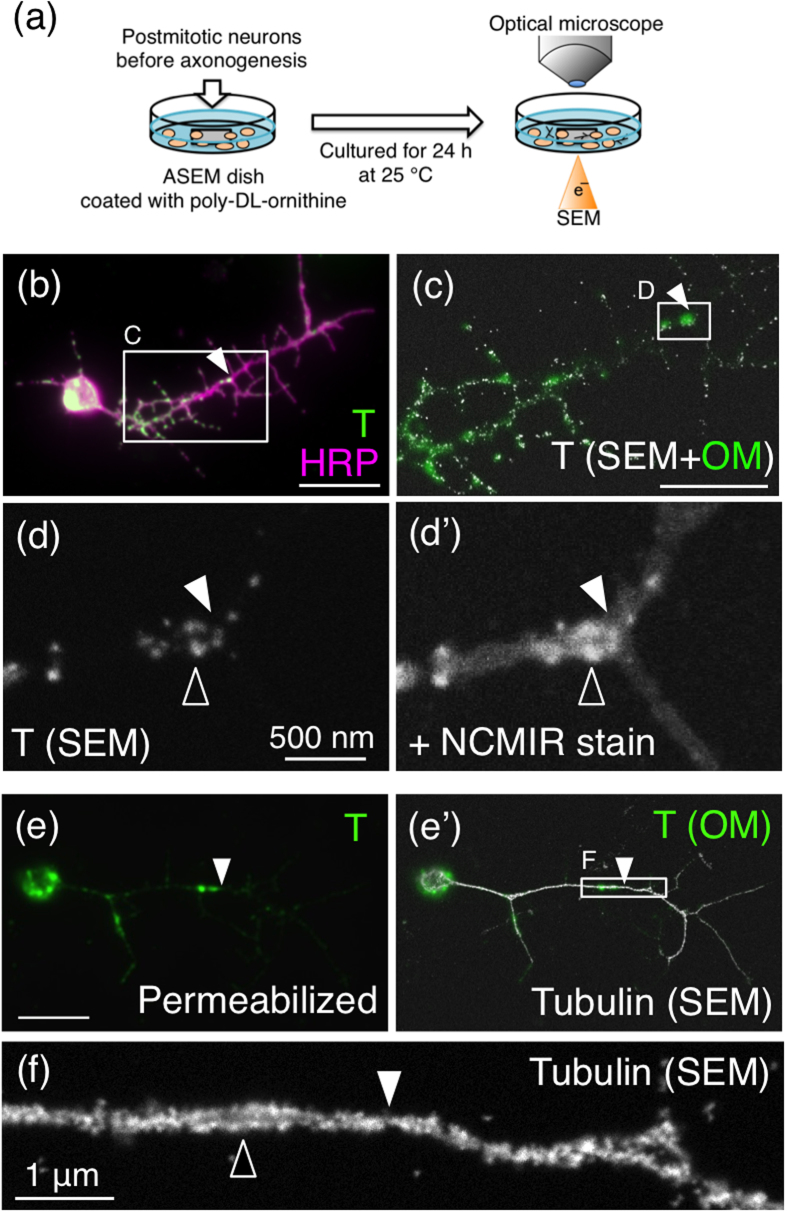
Ultrastructural features characteristic to the intra-axonal boundary. Filled arrowheads indicate the intra-axonal boundary. Wild-type 24-h-cultured primary neurons were analysed. (**a**) Observation of primary cultured neurons using the atmospheric scanning electron microscope (ASEM) and an optical microscope (OM). (**b**) OM image showing the localization of T and HRP antigens on the surface of a neuron. (**c**–**d’**) ASEM images showing the localization of T antigens on the surface of the neuron. (**c**) Merged ASEM (white) and OM (green) images of rectangle C in **b**. T antigens accumulated at the intra-axonal boundary. (**d**) High-magnification ASEM image of rectangle D in **c**. The open arrowhead indicates the characteristic structure of T antigens. (**d’**) Field in d after counter-staining by the modified NCMIR method to visualize the cytoplasm. (**e**) OM image showing localization of T antigens in a permeabilized primary neuron. T antigens are localized in the middle of the axon segment. (**e’**,**f**) ASEM images showing the localization of microtubules in the same neuron. (**e’**) Merged ASEM (white) and OM (green) images of α-tubulins and T antigens, respectively. (**f**) High-magnification ASEM image of rectangle F in e’. The two tubulin bundles (open arrowhead) in the axon are in contact at the intra-axonal boundary (filled arrowhead). Scale bars: 10 μm (**b**,**e**), 5 μm (**c**), 1 μm (**f**), and 500 nm (**d**).

In almost 46% of 24-h cultured neurons, T antigens were localized largely in the middle of the axon surface (Fig. 1h; categorized as “Middle”, see Methods). This suggests that the vesicles containing proteins bearing T antigens were preferentially transported to the intra-axonal boundary. We then observed the ultrastructural localization of T antigens in natural aqueous solution using ASEM (Fig. 2a)^3^. The neurons were cultured on the SiN-film-windowed dish, fixed, and labelled with both fluorescence and Nanogold under non-permeabilizing conditions, and observed by correlative optical microscopy (OM) and inverted scanning electron microscopy (SEM). When T antigens were localized abundantly near the intra-axonal boundary (Fig. 2b,c; filled arrowheads), high-magnification ASEM revealed that they were gathered in a dotted circular pattern (Fig. 2d; open arrowhead). The neurons were counter-stained by the modified National Center for Microscopy and Imaging Research (NCMIR) method^22^ to visualize the surrounding structures in the axon (Fig. 2d’). Because microtubules are used as rails to transport proteins from the soma to axon terminals in neurons^23^, α-tubulin was labelled with gold particles under permeabilizing conditions and visualized by ASEM. The intra-axonal boundary was defined by the position of fluorescence-labelled T antigens (Fig. 2e,e’; filled arrowheads). The intra-axonal boundaries defined by surface and internal T antigens are at the same position (Supplementary Fig. S4). At high magnification, two tubulin bundles were seen to run beside one another (Fig. 2f; open arrowhead), appearing to make contact at least at the intra-axonal boundary and possibly elsewhere (Fig. 2f; filled arrowhead). These characteristic structural features near the intra-axonal boundary might reflect the trafficking of proteins carrying T antigens.

### Localization of Shot and its role in the allocation of T antigens

**Figure 3:**
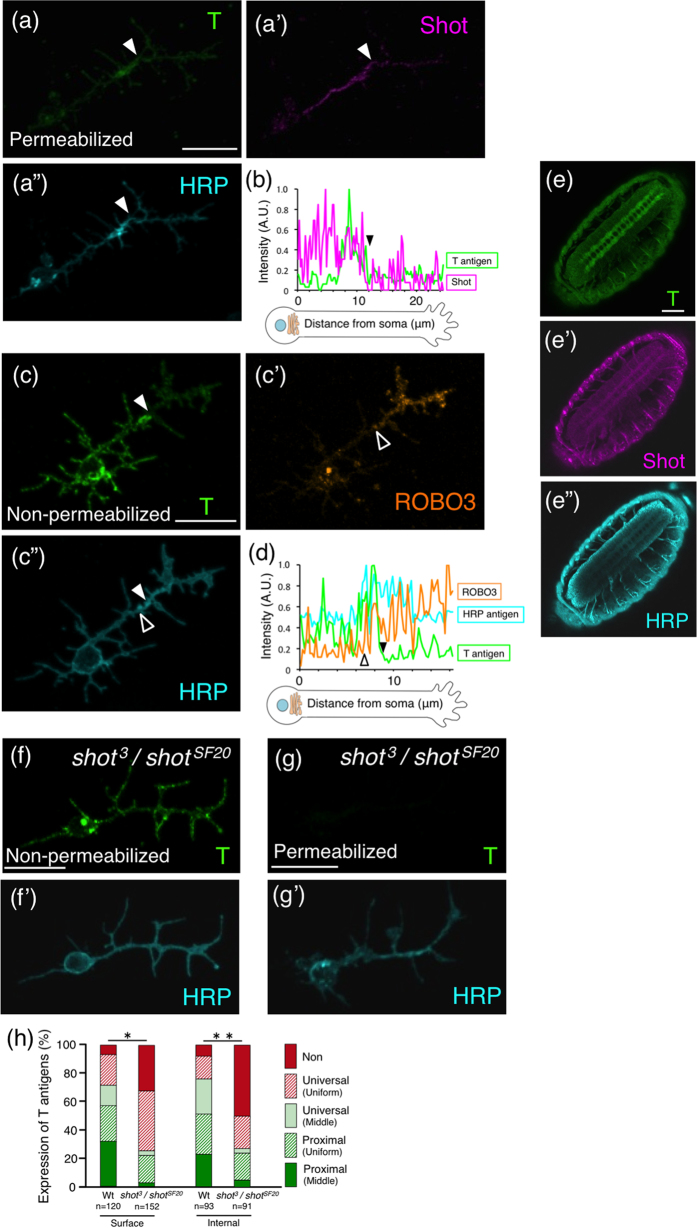
Proximal localization of Shot and its mediation of T antigen trafficking specifically to the proximal segment. (**a**–**a”**) Localization of T antigens, Shot, and HRP antigens in a permeabilized wild-type primary cultured neuron. Similar to T antigens, Shot was localized in the proximal axon segment in 40% of the permeabilized neurons (**a’**). The intra-axonal boundary (filled arrowheads) defined by localization of Shot was consistent with that defined by T antigens. (**b**) Fluorescence intensity of T antigens and Shot along axons. The filled arrowhead indicates the intra-axonal boundary. (**c**–**c”**) Localization of T antigens, ROBO3, and HRP antigens on the surface of a wild-type neuron. T antigens are localized on the surface of the proximal axon segment. Conversely, ROBO3 is localized on the surface of the distal axon segment. Filled and open arrowheads indicate the intra-axonal boundaries defined by the localization of T antigens and ROBO3, respectively (**c**,**d**). (**d**) Fluorescence intensity of T antigens, ROBO3, and HRP antigens along axons. (**e**–**e”**) Localization of T antigens, Shot, and HRP antigens in a wild-type embryo at stage 16. T antigens and Shot were both expressed in the CNS and in the PNS. (**f**,**f’**) Localization of T and HRP antigens on the surface of a *shot*^*3*/^*shot*^*SF20*^ transheterozygous mutant neuron. (**g**,**g’**) Localization of T and HRP antigens in a *shot*^*3*/^*shot*^*SF20*^ mutant permeabilized neuron. (**h**) Histogram showing the localization of T antigens in/on the wild-type and *shot*^*3*/^*shot*^*SF20*^ mutant neurons. The histogram of the surface expression for the wild-type is the same as that at 24 h (shown in Fig. 1h). In the *shot*^*3*/^*shot*^*SF20*^ mutant, the percentage of neurons with proximal localization of T antigens (green) was significantly decreased. *P*-values were calculated using the G-test. **p = 3.3 × 10^−14^, *p = 1.8 × 10^−12^. Scale bar: 10 μm (**a**,**c**,**f**,**g**), 50 μm (**e**).

We analysed mutants of possible candidate *Drosophila* genes involved in the proximal localization of T antigens, and then identified Short stop (Shot), a crosslinker protein that bridges between F-actin and α-tubulin, as a key molecule for their specific localization. We stained primary cultured *Drosophila* neurons and embryos with an anti-Shot antibody under permeabilizing conditions. Shot was localized in proximal axon segments in 40% of the permeabilized neurons (Fig. 3a,b and Table 1). Moreover, the intra-axonal boundary defined by this localization was consistent with the boundary defined by internal T antigens (Fig. 3a,b; filled arrowheads).

ROBO3, which is a member of the axon guidance receptor roundabout (ROBO) family, has been reported to localize to the surface of distal axon segments^1^ (Fig. 3c’,d; open arrowheads). In the present study, the position of the intra-axonal boundary on the surface defined by the localization of T antigens (Fig. 3c,d; filled arrowheads) was subtly different from the position defined by ROBO3 (Fig. 3c’–d; open arrowheads); the proximal axon segment indicated by T antigens slightly overlapped with the distal axon segment defined by ROBO3 (Fig. 3c”,d; filled and open arrowheads). In embryos, Shot was expressed in the CNS and the peripheral nervous system (PNS) at the same level, and co-localized with T antigens (Fig. 3e–e”). Together, these results suggest that Shot functions in the proximal axon segment and is related to the localization of T antigens.

In both *Drosophila* and mammalian neurons, newly synthesized membrane proteins are preferentially inserted at the axon tip (the growth cone) during axonal extension^2^,^24^,^25^. In 70% of wild-type primary neurons, T antigens were localized specifically to the surface of proximal axon segments (Fig. 3h; Surface, green), whereas in 21% of the neurons they were localized on the whole axon (Fig. 3h; Surface, red diagonal line). These results suggest the following possibility. Predominantly, T antigens are carried by vesicles that specifically transport them to proximal axon segments. By a secondary subordinate pathway, the common membrane protein transport pathway, T antigens are also brought to the tip of the axon, resulting in T antigen distribution on the whole axon. In contrast, HRP antigens were localized in/on the whole axon in 100% of the wild-type neurons (Fig. 3a”,c”), indicating that the common membrane protein transport pathway is their dominant trafficking pathway.

To determine whether Shot is involved in the specific localization of T antigens to proximal axon segments, we analysed primary cultured neurons of *shot*^*SF20*^ and *shot*^*3*^ homozygous mutants and a *shot*^*3*^/*shot*^*SF20*^ transheterozygous mutant (Table 2). In more than 90% of permeabilized neurons from *shot* null mutants, Shot was not expressed (Supplementary Fig. S5). The percentage of non-permeabilized neurons with proximal localization of surface T antigens was significantly decreased (Fig. 3h and Table 2; Surface, green and bold, respectively; compare with Table 1), whereas the universal localization of HRP antigen was unchanged and observed in 100% of the *shot* mutant neurons (Fig. 3f’). Thus, localization of T antigens specifically to the proximal axon segments was impaired by *shot* mutation. Moreover, proximal localization of surface T antigens was rescued by neuronal expression of wild-type Shot in the *shot* mutant background (Supplementary Fig. S6), demonstrating that Shot is required for proximal localization of surface T antigens. Further, in *shot* mutant neurons, the percentage of neurons with T antigen localization on the whole axon surface was increased (Fig. 3f,h and Table 2; Surface, red diagonal line and Uniform (plain), respectively). This suggests that inhibition of the dominant transportation pathway led to enhancement of the subordinate pathway that conveys T antigens to the axon tip.

We then examined the intracellular localization of T antigens in permeabilized primary cultured neurons in *shot* mutants. In 74% of wild-type neurons, intracellular T antigens were localized in proximal axon segments (Fig. 3a,h and Table 1; Internal, green and bold, respectively). These results suggest that the transport vesicles containing T antigens are trapped in proximal segments and then transported to the plasma membrane. In *shot* mutant neurons, the percentage of neurons with proximal T antigen localization in the axon was significantly decreased (Fig. 3h; Internal, green). By contrast, the localization of intracellular HRP antigens was unchanged, and HRP antigens were localized in the whole axon in 100% of *shot* mutant neurons (Fig. 3a”,g’). Moreover, proximal localization of internal T antigens was rescued by neuronal expression of wild-type Shot in the *shot* mutant background (Supplementary Fig. S6). These results demonstrate that the trafficking restriction for transport vesicles containing T antigens at the boundary was inhibited by *shot* mutation. In agreement with these findings, the percentage of neurons with universal localization of intracellular T antigens was increased in *shot* mutant neurons (Fig. 3h and Table 2; Internal, red diagonal line and Uniform (plain), respectively). Taken together, these results suggest that in wild-type neurons, transport vesicles containing T antigens are trapped at proximal axon segments and transported to the nearby plasma membrane in a Shot-dependent manner, whereas in *shot* mutant neurons the vesicles are distributed to the whole axon and not trapped at proximal axon segments.

### Shot mutation does not affect the synthesis of T antigens

It has been reported that Shot controls translation through binding with the translation inhibitor Krasavietz (also known as Kra, Exba), and that together Kra and Shot control midline axon repulsion^18^. Thus, a Kra–Shot complex might regulate the translational expression of T antigens. We therefore examined the possibility of translational alteration by Shot mutation. To determine the total amounts of T antigens in *shot* null mutant embryos, we performed PNA lectin and Western blot analyses of the lysate of the embryos. The expression of T antigens by the wild-type and *shot* mutant embryos did not differ significantly (Supplementary Fig. S7). Taken together, the results show that the Shot mutation does not affect the synthesis of T antigens, but is involved in their localization.

### Shot is involved in tubulin bundle formation

**Figure 4:**
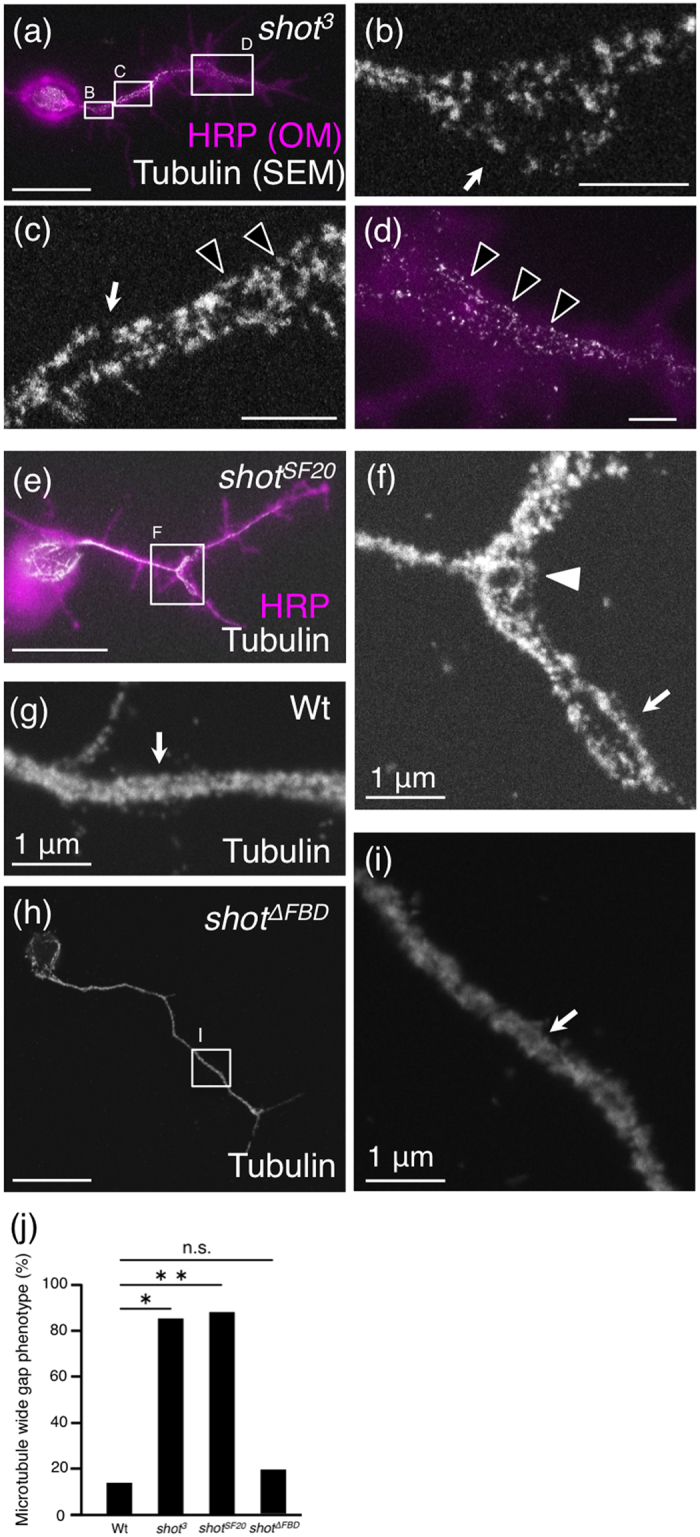
Shot is involved in bundle formation of tubulin in primary cultured neurons. (**a**–**f**) ASEM images showing the localization of α-tubulin in permeabilized primary cultured neurons of the *shot*^*3*^ (**a**–**d**) and *shot*^*SF20*^ (**e**,**f**) mutants. Arrows and arrowheads indicate defects in microtubule bundles. (**a**,**d**,**e**) Merged ASEM (white) and OM images (magenta) of the α-tubulin and HRP antigen distributions, respectively. (**b**–**d**) High magnification of rectangles B, C, and D in **a**. (**f**) High magnification ASEM image of the rectangle F in **e**. (**g**) ASEM image showing the localization of α-tubulin in permeabilized primary-cultured wild-type neurons. (**h**,**i**) ASEM images showing the localization of α-tubulin in the permeabilized primary cultured *shot*^*ΔFBD*^ (*shot*^*k03405*^) mutant, which expresses only Shot protein lacking the F-actin binding domain. (**i**) High magnification of rectangle I in **h**. (**j**) Histogram showing the percentage of abnormalities in microtubule formation. All neurons with a spread-out morphology of microtubules at more than one site in axon were classified as having abnormal tubulin bundle formation. In more than 85% of *shot* null mutant (*shot*^*SF20*^ and *shot*^*3*^) axons, microtubules displayed a spread-out morphology, whereas this phenotype was observed in only 19% of *shot*^*ΔFBD*^ mutant axons. The percentage of neurons showing abnormalities and the number of observations for each genotype are as follows: wild-type, 13.0, *n* = 23; *shot*^*3*^, 85.1, *n* = 27; *shot*^*SF20*^, 88.0, *n* = 25; and *shot*^*ΔFBD*^, 19.2, *n* = 26. *P*-values were calculated using the G-test. **p = 3.7 × 10^−8^, *p = 8.1 × 10^−8^, and n.s = 0.56. Scale bar: 10 μm (**a**,**e**,**h**), 1 μm (**b**–**d**,**f**,**g**,**i**).

To investigate morphological features in *shot* mutants neurons, we observed the ultrastructure of microtubules by ASEM (Fig. 4a–f). In *shot*^*3*^ mutant neurons, the tubulin bundles displayed a spread-out morphology (Fig. 4b; arrow), were disconnected (Fig. 4c; arrow), and had several gaps (Fig. 4c,d; open arrowheads). In *shot*^*SF20*^ mutant neurons, the tubulin bundles formed a loop (Fig. 4f; filled arrowhead) and the two tubulin bundles were further apart than in the wild-type (compare Fig. 4f with Fig. 4g; arrow). In more than 85% of mutant axons, tubulin bundles had showed a spread-out morphology as mentioned above (Fig. 4j). These results demonstrate that Shot is involved in the formation of tubulin bundles in primary cultured neurons. Consistent with these results, previous a light microscopic study reported that microtubules in *shot* mutant *Drosophila*, neurons display irregular trajectories and frequent loops^19^.

### F-actin binding domain of Shot is required for the proximal localization of T antigens

We predicted that Shot might interact and function with F-actin near the intra-axonal boundary, as we have reported that F-actin accumulates near the intra-axonal boundary^3^. To examine this possibility, we analysed neurons derived from the *shot*^*ΔFBD*^ mutant, which expresses only Shot protein lacking the F-actin binding domain. Surprisingly, abnormalities of tubulin bundle formation were observed in more than 85% of *shot* null mutant (*shot*^*3*^ and *shot*^*SF20*^) axons (Fig. 4a–f) but found in only 19% of *shot*^*ΔFBD*^ mutant axons by ASEM (Fig. 4j; compare with wild-type). The ratio of abnormalities in *shot*^*ΔFBD*^ mutant axons was almost the same as that in wild-type axons. No severe abnormalities such as those in *shot* null mutant (Fig. 4a–f) were observed in *shot*^*ΔFBD*^ mutant and wild-type axons. Furthermore, 81% of *shot*^*ΔFBD*^ mutant neurons displayed normal tubulin bundle formation; for example, the distance between two tubulin bundles in *shot*^*ΔFBD*^ mutant axons was similar to that in wild-type axons (compare Fig. 4i with Fig. 4g; arrow). These results demonstrate that tubulin bundles form normally in *shot*^*ΔFBD*^ mutant axons.

**Figure 5:**
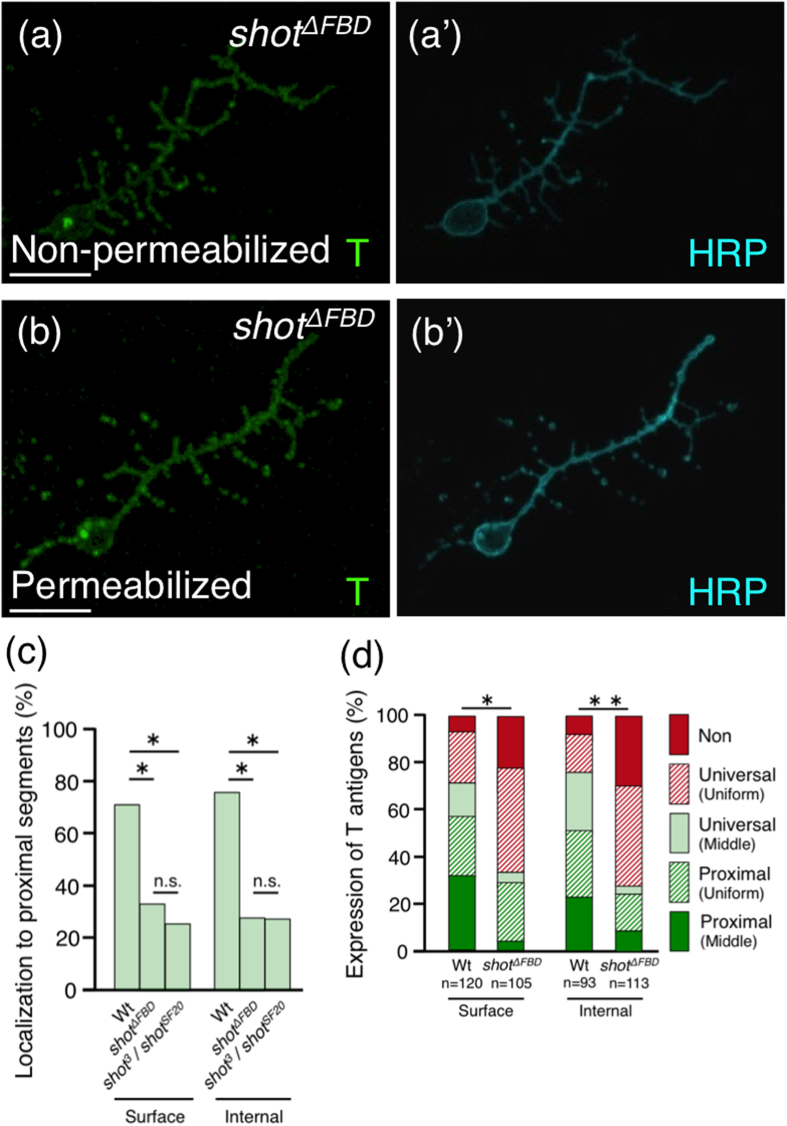
F-actin binding domain of Shot is required for proximal localization of T antigens. (**a**,**a’**) Localization of T and HRP antigens on the surface of a *shot*^*ΔFBD*^ mutant non-permeabilized neuron, which expresses only Shot protein lacking F-actin binding domain. (**b**,**b’**) Localization of T and HRP antigens in a *shot*^*ΔFBD*^ mutant permeabilized neuron. (**c**) Histogram showing the percentage of neurons with proximal localization of T antigens in/on the wild-type, *shot*^*ΔFBD*^ mutant, and *shot*^*3*/^*shot*^*SF20*^ mutant neurons. Compared to the wild-type neurons, both permeabilized and non-permeabilized *shot*^*ΔFBD*^ mutant neurons showed a significantly decrease in the percentage of neurons with proximal T antigen localization in the axon, to the same level as observed in the *shot*^*3* /^*shot*^*SF20*^ mutant neurons. *P*-values were calculated using the G-test. *p < 1.0 × 10^−7^, n.s. >0.1. (**d**) Histogram showing the localization of T antigens in/on the wild-type and *shot*^*ΔFBD*^ mutant neurons. In the *shot*^*ΔFBD*^ mutant, the percentage of neurons with T antigen localization in/on the whole axon (Universal (Uniform)) was significantly increased. The histogram for the wild-type is the same as that shown in Fig. 3h. *P*-values were calculated using the G-test. **p = 6.0 × 10^−11^, *p = 5.7 × 10^−9^. Scale bar: 10 μm (**a**,**b**).

We then analysed the localization of T antigens in *shot*^*ΔFBD*^ mutant neurons. Compared to the wild-type neurons, both permeabilized and non-permeabilized *shot*^*ΔFBD*^ mutant neurons showed a significant decrease in the percentage of neurons with proximal T antigen localization in the axon, to the same level as observed in the *shot* null mutant (Fig. 5a–c and Table 2; bold); however, the *shot*^*ΔFBD*^ mutant axons displayed normal formation of tubulin bundles (Fig. 4h–j). Similarly, the percentage of neurons with universal localization of T antigens was increased (Fig. 5d and Table 2; red diagonal line and Uniform (plain), respectively) in both permeabilized and non-permeabilized *shot*^*ΔFBD*^ mutant neurons. These results clearly demonstrate that the F-actin binding domain of Shot is required for the proximal localization of T antigens.

## Discussion

Herein, we clarify part of the mechanism underlying the preferential proximal localization of T antigen, mucin-type core 1 *O*-glycan (Fig. 1a), for the first time. T antigen is localized specifically in/on proximal axon segments (Figs 1b,h and 3a,h, and Table 1). By using ASEM, we found that T antigens and microtubules form characteristic structures near the intra-axonal boundary (Fig. 2b–f). Moreover, we newly identified Shot as a mediator of the proximal localization of T antigen; in *shot* null mutant neurons, the preferential localization of T antigen to proximal segment was inhibited (Fig. 3f–h and Table 2) and rescued by neuronal expression of wild-type Shot (Supplementary Fig. S6). The F-actin binding domain of Shot was required for proximal localization (Fig. 5a–d and Table 2). Taken together, these results clearly demonstrate that Shot mediates the proximal localization of T antigen through binding to F-actin.

**Figure 6:**
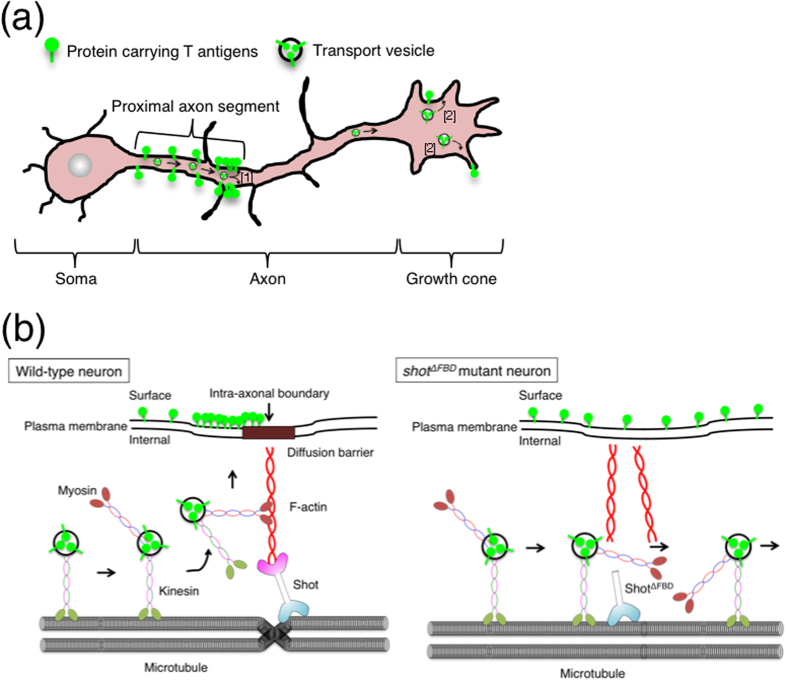
Schematic diagram of axonal trafficking of the membrane proteins carrying T antigens. (**a**) Schematic representation of the trafficking pathways of the membrane proteins carrying mucin-type core 1 *O*-glycan T antigens. This study suggests that trafficking of T antigens in an axon takes place by the following two pathways: [1] a dominant pathway, where vesicles containing the membrane proteins carrying T antigens are specifically transported to proximal axon segments, and [2] a subordinate pathway, where T antigens are transported to the growth cone. (**b**) Proposed detailed scheme for the dominant pathway of T antigen transport to proximal axon segments. In wild-type neurons (left), transport vesicles containing the membrane proteins carrying T antigens are transferred to kinesins on microtubules and are transported from the soma towards the growth cone. On reaching the point where two tubulin bundles are in contact and are linked to F-actin by Shot, vesicles are detached from the microtubules and are transferred to F-actin. Myosins then further transport these vesicles on F-actin to the plasma membrane of the proximal axon region. After fusion with the plasma membrane, the membrane proteins carrying T antigens in the intra-axonal boundary diffuse within the surface of the proximal segment. In the *shot*^*ΔFBD*^ mutant (right), Shot cannot bind to F-actin; therefore, the vesicles may transit through the position where Shot^ΔFBD^ localizes, without being transferred to F-actin.

In nearly 50% of the wild-type neurons, T antigens were localized highly in/on the middle region of an axon (Fig. 3h and Table 1; sum of “Universal (Middle)” and “Proximal (Middle)”), which is the region near the intra-axonal boundary in the proximal axon segment. In *shot*^*ΔFBD*^ and also *shot* null mutants, the percentage of neurons with boundary-rich localization of T antigens (categorized as “Universal (Middle)” or “Proximal (Middle)”) was more markedly decreased than the percentage of neurons categorized as “Proximal (Uniform)” for both permeabilized and non-permeabilized neurons (Figs 3h and 5d, and Table 2; compare sum of “Universal (Middle)” and “Proximal (Middle)” with “Proximal (Uniform)”). In addition, we have reported that F-actin accumulates near the intra-axonal boundary^3^. These results suggest that the F-actin binding domain of Shot is involved in the transport of T antigens, especially to the region near the intra-axonal boundary. Furthermore, the proximal localization of BP102 antigen, which is a *Drosophila* CNS marker, was also impaired in *shot* mutant neurons (Table 2). Based on these results, we proposed the following proximal transport mechanism: Shot binds to F-actin, crosslinks F-actin and microtubules at the intracellular region near the intra-axonal boundary, and achieves the localization of T antigens and BP102 antigens specifically to the proximal axon segment (Fig. 6a,b).

Most T antigens in the lysate of whole embryos are derived from neurons, as in *Drosophila* embryos T antigens are mainly expressed in neurons defined by expression of neuronal marker HRP antigens (Supplementary Fig. S1). In addition, PNA lectin blot showed that the amount of T antigens in the lysate of whole embryo was not different between the wild-type and the *shot* null mutant, namely the *shot*^*SF20*^ mutant (Supplementary Fig. S7). These results showed that the expression level of T antigen is not changed in *shot* mutant neurons. However, in *shot* mutants, the percentage of neurons with undetectable levels of T antigen was increased (Fig. 3h and Table 2; “Non”). It is considered that one possible reason for this increase is the inhibition of T antigen’s transport by abnormal organization of microtubules. Severely abnormal organization of microtubules (Fig. 4a–f) was detected in *shot* null mutant neurons but not *shot*^*ΔFBD*^ mutant neurons by ASEM, and might have caused the decrease in T antigens (Fig. 3h and Table 2; “Non”). Thus, in *shot* null mutants, the trafficking of transport vesicles containing T antigens might be impaired, resulting in a smaller amount of T antigens on the axon. Consistent with these results, the percentage of neurons with undetectable levels of surface or internal T antigens in the *shot*^*ΔFBD*^ mutant was less than that in *shot* null mutants (Fig. 3h compare with Fig. 5d; “Non”; Table 2). However, even the levels in the *shot*^*ΔFBD*^ mutant were not equal to those in the wild-type; the mutant still showed an increase (Fig. 5d; “Non”; Table 2). In both *shot* null mutant and *shot*^*ΔFBD*^ mutant neurons, the percentage of neurons with T antigen localization in/on the whole axon was increased (Figs 3h and 5d, and Table 2; “Universal (Uniform)”). Therefore, it is considered that the density of the T antigen decreased to half in the localization in/on the whole axon, resulting in reduction of T antigen fluorescence in/on axons and an increased amount of neurons with undetectable levels of surface or internal T antigen in *shot* mutants. These two mechanisms might function synergistically and cause an increase in the undetectable levels of T antigen in *shot* mutants.

Laminin B2, which is a major component of the extracellular matrix, has been reported as a protein that carries T antigens in *Drosophila* neurons^15^,^26^. However, it is a secreted protein. Previous reports suggested that in mammals, α-dystroglycan, which is a central component of the dystrophin-glycoprotein complex, also carries T antigens^27^. Our lectin blot analyses showed that PNA recognized a large number of proteins including a band corresponding to molecular weight of *Drosophila* dystroglycan. Therefore, we expect that *Drosophila* dystroglycan also carries T antigens and is localized specifically in proximal axon segments, similar to T antigens. This hypothesis is currently under investigation.

To examine whether reduction in T antigens influences the proximal localization of BP102 antigens, we analysed the localization of BP102 antigens in *C1*β*3GalT1* mutant neurons. BP102 antigens were still localized at the proximal axon segments in *C1*β*3GalT1* mutant neurons, revealing that T antigens were not involved in proximal localization of BP102 antigens (Supplementary Fig. S8). However, it is still unknown whether proximal localization of T antigens is achieved by T antigens themselves or by T antigen-carrying proteins. If T antigens themselves contribute to proximal localization, T antigens should control the proximal localization of all proteins carrying T antigens. Alternatively, if T antigen-carrying proteins play a key role in proximal localization, the common functional domain required for proximal localization should exist among these proteins. The examination of these possibilities was the next step in this study.

Shot possesses diverse functional domains, namely, an F-actin binding domain (first calponin domain), a plakin domain, a spectrin repeats domain, two putative Ca^2+^-binding domains (EF-hand domain), and a microtubule-binding domain (Gas2 domain)^16^. Various roles of the individual domains in neurons have been reported: axon extension^16^ and midline axon repulsion^18^ are mediated by the calponin domain; the localization of the cell adhesion molecule Fasciclin2 and axonal growth are mediated by the plakin domain^28^; the EF-hand domain is the binding site for Krasavietz, a translation inhibitor^18^; and microtubule organization is regulated via the calponin and Gas2 domains^19^. We could find no difference in formation of microtubule between *shot*^*ΔFBD*^ mutant and wild-type axons (Fig. 4g,i,j), although abnormality of microtubule organization in *shot*^*ΔFBD*^ mutant axons was mentioned in a previous report^19^. It might be caused by the different culture conditions used in each experiment; (i) cells were grown with culture medium containing serum in our experiment and with serum-free medium in the previous experiment, and (ii) cells were grown on poly-DL-ornithine-coated glass in our experiment and on non-coated glass or concanavalin A-coated glass in the previous experiment. Our condition might enable us to analyse the function of the F-actin binding domain of Shot without defective microtubule organization. In this condition, we showed that the F-actin binding domain of Shot is required for proximal localization of T antigen (Fig. 5a–d and Table 2). Moreover, T antigen accumulated in the intracellular region near the intra-axonal boundary in wild-type neurons, whereas it rarely localized there in *shot*^*ΔFBD*^ mutant neurons (Fig. 5d and Table 2; “Middle”). These results demonstrate that F-actin binding domain is also required for accumulation of T antigens near the intra-axonal boundary as well as proximal localization of T antigens.

*Drosophila* has a single spectraplakin, Shot, whereas two types of spectraplakins, BPAG1 and MACF1, exist in mammals. These molecules possess high sequence homology and share the same domain structure^29^,^30^; several functions of spectraplakin have been reported to be evolutionarily conserved between *Drosophila* and mammals^19^,^31^. Several reports have revealed that human spectraplakins are involved in neurological diseases. A new hereditary sensory and autonomic neuropathy (type VI) has been reported to be caused by a homozygous mutation in the *BPAG1* gene^32^,^33^. Recently, it was also reported that the *MACF1* gene is a strong candidate susceptibility gene for schizophrenia, based on the identification of *de novo* mutations in schizophrenic patients^34^. However, the molecular mechanisms involved are largely unknown. Because intra-axonal compartmentalization is evolutionarily conserved between *Drosophila* and mammals^1^,^2^, the novel function of spectraplakin demonstrated in this study, i.e., the mediation of the *O*-glycan trafficking that targets some molecules to proximal axon segments, might be conserved in mammals and provide a novel clue helping to elucidate the pathogenic mechanisms of neurological diseases.

## Methods

### *Drosophila* strains

*Drosophila* mutants, i.e., *shot*^*3*^, *shot*^*SF20*^, and *shot*^*k03405*^ (*shot*^*ΔFBD*^), from Bloomington Stock Center were used in the experiments. The Canton-S strain was used as the wild-type for the analyses. It has been suggested that both *shot*^*SF20*^ and *shot*^*3*^ are the null allele^17^,^28^,^35^. The *shot*^*ΔFBD*^ mutant produces only endogenous isoforms lacking the F-actin binding domain^19^.

### Primary cell cultures

We used a previously described method to obtain primary cell cultures^1^,^36^. *Drosophila* embryos at stages 9–11 were collected and homogenized in Schneider’s *Drosophila* medium (Gibco) containing 2% foetal bovine serum (FBS; Gibco). The mixture was then treated with 0.05% trypsin for 1 min. Subsequently, the cells, including postmitotic neurons before axonogenesis, were suspended in Schneider’s medium containing 10% FBS and cultured at 25 °C for 12–24 h or 21 d without additional CO_2_ on 35-mm glass bottom dishes (MatTek) or standard 35-mm bio-ASEM dishes (JEOL, Ltd.) that had previously been coated with 1 mg/ml poly-DL-ornithine (Sigma). The cell density was adjusted to prevent cell–cell interactions. The neurons were cultured for 24 h except when indicated otherwise (Fig. 1 and Supplementary Fig. S3).

### Immunostaining, lectin staining, and Nanogold labelling

Immunostaining, lectin staining, and FluoroNanogold labelling of primary cultured cells were performed as described previously^3^. Immunostaining of whole embryos at stage 16 was also performed as described previously^6^. Briefly, fixed cells and embryos were treated with Block Ace (Dainihon Pharmaceutical) to block nonspecific binding; to observe intracellular molecules, the cells were permeabilized with 0.1% Triton X-100 (Sigma)/phosphate-buffered saline (PBS) before blocking. For primary labelling, the cells were incubated with PNA-biotin (1:100; Seikagaku) or antibodies in 10% Block Ace/PBS. The following primary antibodies were used: rabbit anti-HRP (1:1000; MP Biomedicals), mouse anti-Robo3 extracellular (1:10; Developmental Studies Hybridoma Bank [DSHB]), mouse anti-Shot (1:10; DSHB), mouse BP102 anti-CNS axons (1:10; DSHB), rabbit anti-α-tubulin (1:200; MBL), and mouse anti-α-tubulin antibodies (1:200; Sigma). Alexa Fluor 555-conjugated streptavidin (1:300; Life Technologies) and secondary antibodies, i.e., Alexa Fluor 488-conjugated goat anti-mouse IgG and Cy5-conjugated goat anti-rabbit IgG antibodies (1:300; Life Technologies), were used for fluorescence labelling. Streptavidin doubly conjugated with 1.4 nm Nanogold and Alexa Fluor 594 (1:1000; Nanoprobes) and Fab’ anti-rabbit IgG doubly conjugated with 1.4 nm Nanogold and Alexa Fluor 488 (1:500; Nanoprobes) were used for FluoroNanogold labelling. After imaging by fluorescence microscopy, cells were fixed with 1% glutaraldehyde to crosslink the antibodies. Enhancement of the Nanogold particles for ASEM observation in solution was performed using GoldEnhance EM (Nanoprobes).

### Modified NCMIR staining

Neurons observed by SEM were counter-stained using the modified NCMIR method^14^,^22^,^37^. Briefly, neurons were washed with 0.15 M cacodylate buffer (CB, pH 7.4) containing 2 mM CaCl_2_, and fixed and stained with the same buffer supplemented with 1.5% potassium ferricyanide (Sigma) and 2% aqueous osmium tetroxide (OsO_4_; Nisshin EM) at 25 °C for 10 min. After washing with deionized distilled water (DDW), neurons were then incubated with filtered 1% thiocarbohydrazide (TCH; Tokyo Chemical Industry) at 25 °C for 20 min. After rinsing with DDW, the neurons were further fixed and stained with 2% aqueous OsO_4_ at 25 °C for 5 min, rinsed with DDW, stained with 2% UA in DDW, and incubated at 4 °C overnight. Finally, after washing with DDW, neurons were stained with 0.4% lead citrate (TAAB Laboratories Equipment) at 25 °C for 2 min.

### Imaging

An LSM 700 confocal microscope (Carl Zeiss) was used for optical microscopy. EM images were taken on a ClairScope ASEM system (JASM-6200, JEOL Ltd). The neurons fixed on the ASEM dish were immersed in 10 mg/ml D-glucose or ascorbic acid in DDW and observed using the inverted SEM of the ASEM. The acceleration voltage of the SEM was 20 kV, and backscattered electrons (BSE) from the specimens were recorded by a BSE imaging detector to visualize the sample (Fig. 2). The electron dose at the highest magnification of 10,000× was 3.2 e^−^/Å^2^, which is less than 10% of the dose permitted in low-dose cryoelectron microscopy aiming at atomic resolution single particle reconstructions. The images were processed using Adobe Photoshop.

### Classification of neurons

The fluorescence images of neurons were obtained at an 8-bit depth, and the maximum pixel intensity obtained for each label, i.e., for T antigen, BP102 antigen, or Shot, was linearly confined such that the maximum limit of the dynamic range was not exceeded. Fluorescence intensity in an axon was quantified using the ImageJ plug-in, NeuronJ (Imagescience). The whole axon shape was traced using the fluorescence signal of the universally distributed HRP antigen. The intensity of each label was normalized such that 1.0 represented the maximum intensity. If the maximum intensity of a label along the trace was less than 10% of the maximum value of the dynamic range, the neuron was classified as “Non”, which indicated that it was below the limit of detection. Neurons classified as “Non” were not subjected to statistical tests. The others neurons were classified into three groups by the following statistical analysis. The distribution of each label was assessed by a statistical significance test that compares the intensity of the proximal and distal halves of an axon. If the *p*-value was greater than 1.0 × 10^−3^ (Wilcoxon signed rank test), the neuron was classified into the “Universal” distribution category, because no significant difference was observed between the proximal and distal distribution. The other neurons were classified into the “Proximal” or “Distal” distribution categories, depending on whether the particular label was distributed proximally or distally, respectively. Neurons where the label was highly localized in the middle region of an axon were additionally classified into the “Middle” distribution category by visual assessment. The other neurons were classified into the “Uniform” category. Altogether, we classified the observed neurons in terms of the localization of each label into six groups, i.e., Non, Universal (Uniform), Universal (Middle), Proximal (Uniform), Proximal (Middle), and Distal, as illustrated in Fig. 1g. Furthermore, labels categorized as Proximal (Middle), Proximal (Uniform), and Universal (Middle) were all considered to localize specifically to the “proximal axon segment”, because this segment includes the whole area from the cell body to the middle of the axon.

### Statistical analysis

Statistical evaluation of the differences between the groups was performed using the Wilcoxon signed-rank test, G-test, and Dunnett’s test using R software version 3.2.2. *p* < 0.05 was considered significant.

## Additional Information

**How to cite this article**: Kinoshita, T. *et al*. Short stop mediates axonal compartmentalization of mucin-type core 1 glycans. *Sci. Rep.* **7**, 41455; doi: 10.1038/srep41455 (2017).

**Publisher's note:** Springer Nature remains neutral with regard to jurisdictional claims in published maps and institutional affiliations.

## Supplementary Material

Supplementary Information

## Figures and Tables

**Table 1:** Localization of labels in/on wild-type primary cultured neurons

Labels	Number	Category of localization (%)							
		Proximal		Universal		Non	Distal	Total	
		Middle	Uniform	Middle	Uniform			Bold	Plain
T antigen (Surface)	123	**31.8**	**24.4**	**13.8**	21.1	6.5	2.4	**70.0**	30.0
T antigen (Internal)	96	**22.9**	**27.1**	**24.0**	15.6	7.3	3.1	**74.0**	26.0
BP102 antigen (Surface)	82	**34.2**	**20.7**	**13.4**	13.4	15.9	2.4	**68.3**	31.7
BP102 antigen (Internal)	96	**28.2**	**25.0**	**21.9**	8.3	15.6	1.0	**75.1**	24.9
Shot (Internal)	113	**3.5**	**34.5**	**1.8**	45.1	13.3	1.8	**39.8**	60.2
**Localization in/on “Proximal axon segment”**									

Each label was localized specifically in “proximal axon segments” in neurons of following three groups (bold): Proximal (Middle), Proximal (Uniform), and Universal (Middle). On the other neurons it was considered that localization of each label was not confined to “proximal axon segments” (plain).

**Table 2:** Localization of labels in/on *shot* mutants primary cultured neurons

Strain	Labels	Category of localization (%)								
		Proximal		Universal		Uniform	Non	Distal	Total	
		Number	Middle	Uniform	Middle				Bold	Plain
shot^3^/shot^SF20^	T antigen (Surface)	156	**3.2**	**18.5**	**3.2**	40.4	32.1	2.6	**24.9**	75.1
	T antigen (Internal)	91	**5.5**	**18.7**	**3.3**	22.0	50.5	0.0	**27.5**	72.5
	BP102 antigen (Surface)	156	**4.5**	**24.3**	**2.6**	32.1	34.6	1.9	**31.4**	68.6
	BP102 antigen (Internal)	91	**9.9**	**15.4**	**0.0**	11.0	63.7	0.0	**25.3**	74.7
shot^SF20^	T antigen (Surface)	108	**2.7**	**14.8**	**1.9**	41.7	36.1	2.8	**19.4**	80.6
	T antigen (Internal)	78	**0.0**	**28.2**	**0.0**	33.3	38.5	0.0	**28.2**	71.8
	BP102 antigen (Surface)	83	**2.4**	**15.7**	**3.6**	50.6	27.7	0.0	**21.7**	78.3
	BP102 antigen (Internal)	78	**0.0**	**17.9**	**0.0**	17.9	64.2	0.0	**17.9**	82.1
shot^3^	T antigen (Surface)	143	**7.6**	**18.2**	**9.1**	45.5	16.1	3.5	**34.9**	65.1
	T antigen (Internal)	57	**8.7**	**8.8**	**1.8**	22.8	56.1	1.8	**19.3**	80.7
	BP102 antigen (Surface)	145	**10.3**	**21.4**	**6.2**	40.0	21.4	0.7	**37.9**	62.1
shot^ΔFBD^	T antigen (Surface)	111	**4.5**	**23.4**	**4.5**	41.5	20.7	5.4	**32.4**	67.6
	T antigen (Internal)	118	**9.3**	**14.4**	**3.4**	40.7	28.0	4.2	**27.1**	72.9
	BP102 antigen (Surface)	111	**6.3**	**32.4**	**3.6**	43.3	11.7	2.7	**42.3**	57.7
	BP102 antigen (Internal)	118	**13.6**	**12.7**	**0.0**	11.0	60.2	2.5	**26.3**	73.7
**Localization in/on "Proximal axon segment"**										

shot^3^/shot^SF20^: transheterozygous mutant. shot^SF20^, shot^3^: homozygous mutants. shot^ΔFBD^: F-actin binding domain deletion mutant. Each label was localized specifically in “proximal axon segments” in neurons of following three groups (bold): Proximal (Middle), Proximal (Uniform), and Universal (Middle). On the other neurons it was considered that localization of each label was not confined to “proximal axon segments” (plain).
