# Supplementary material for: Short stop mediates axonal compartmentalization of mucin-type core 1 glycans
Source: Sci Rep. 2017 Feb 2;7:41455. doi: 10.1038/srep41455 (PMC5288716; doi:10.1038/srep41455)
Supplement: Supplementary Information [file srep41455-s1.pdf]

Supplementary information

Short stop mediates axonal compartmentalization of mucin-type core 1 glycans

Takaaki Kinoshita[1], Chikara Sato[2], Takashi J. Fuwa[1], and Shoko Nishihara[1*]

[1]Laboratory of Cell Biology, Department of Bioinformatics, Graduate School of Engineering, Soka University, 1-236 Tangi-machi, Hachioji-shi, Tokyo 192-8577, Japan.
[2]Biomedical Research Institute, National Institute of Industrial Science and Technology (AIST) Tsukuba Central 6 and 2, 1-1-1 Higashi, Tsukuba-shi, Ibaraki 305-8566, Japan.

***Corresponding author**
Correspondence and requests for materials should be addressed to S.N.
(E-mail) shoko@soka.ac.jp

Supplementary Methods

Drosophila strains

The Canton-S strain was used as the wild-type for the analyses. *Drosophila* mutants, i.e., *C1β3GalT1^{EY13370} and C1β3GalT1$^{2.1}$*, from Bloomington Stock Center were used in the experiments. *C1β3GalT1$^{2.1}$* was previously described as a null allele[1]. The *C1β3GalT1^{EY13370}* / *C1β3GalT1$^{2.1}$* transheterozygous mutant (described as *C1β3GalT1^{-}*) was used in the experiments. The following strains were used for the rescue experiment: elav-Gal4 and UAS-Shot-full-length from Bloomington Stock Center.

Western blot and lectin blot analyses

Embryos at stage 16 were collected and homogenized in 50 mM Tris–HCl (pH 7.5), 150 mM NaCl, and 1 % Triton X-100 with protease inhibitors (1 mM PMSF, 10 µg/mL aprotinin, 10 µg/mL pepstatin A, 10 µg/mL leupeptin, and 1 µg/mL antipain), and debris was removed by centrifugation at 18,000 *g* for 10 min before analysis by lectin or Western blotting. The embryonic extracts were separated by 6.0 % sodium dodecyl sulphate-polyacrylamide gel electrophoresis and transferred onto polyvinylidene difluoride membranes (Millipore). For the lectin blot analyses, the membranes were incubated with an HRP-conjugated PNA lectin (1:10000; Seikagaku). For Western blot analyses, the membranes were incubated with mouse BP102 anti-CNS axons (1:100; DSHB) antibody or a mouse anti-α-tubulin antibody (1:2000; Sigma), blocked, washed, and incubated further with an HRP-conjugated anti-mouse IgG antibody (1:20000; Cell Signaling Technology). Bands were visualized using the ECL prime Western blotting detection reagent (GE Healthcare Bio-Science Japan).

Observing the localization of surface BP102 antigens and internal T antigens in the same neurons

Immunostaining for surface BP102 antigens on the primary cultured neurons was performed as described in the Methods in the main body of text. After surface BP102 antigens were observed, the neurons were permeabilized with 0.1 % Triton X-100 (Sigma)/phosphate-buffered saline (PBS), treated with Block Ace (Dainihon Pharmaceutical), and stained by PNA-biotin (1:100; Seikagaku) and Alexa Fluor 555-conjugated streptavidin (1:300; Life Technologies) as described in the Methods in the main body of text.

Kinoshita *et al*. Supplementary Figure 1

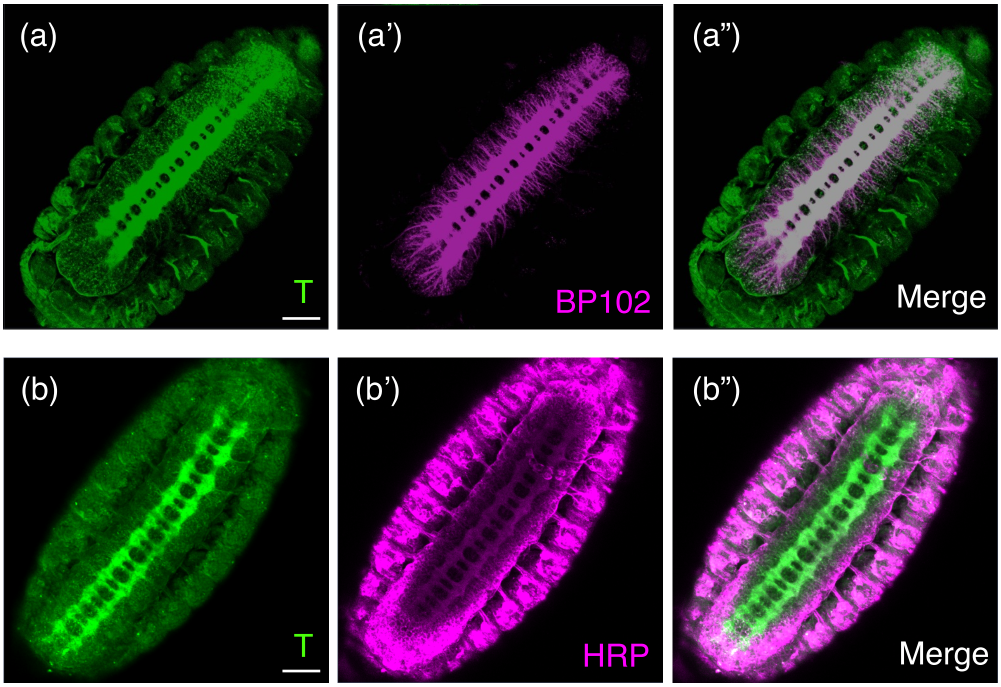

Figure S1 | Localization of T, BP102, and HRP antigens in wild-type embryos

(a-a") Localization of T antigens (green) and BP102 antigens (magenta) in the embryo at stage 16. T antigens are co-localized with the CNS marker, BP102 antigens. (b-b") Localization of T antigens (green) and HRP antigens (magenta) in the embryo at stage 16. T antigens are expressed in neurons defined by the expression of the neuronal marker, HRP antigen.

Scale bar: 50 μm (a, b).

Kinoshita *et al*. Supplementary Figure 2

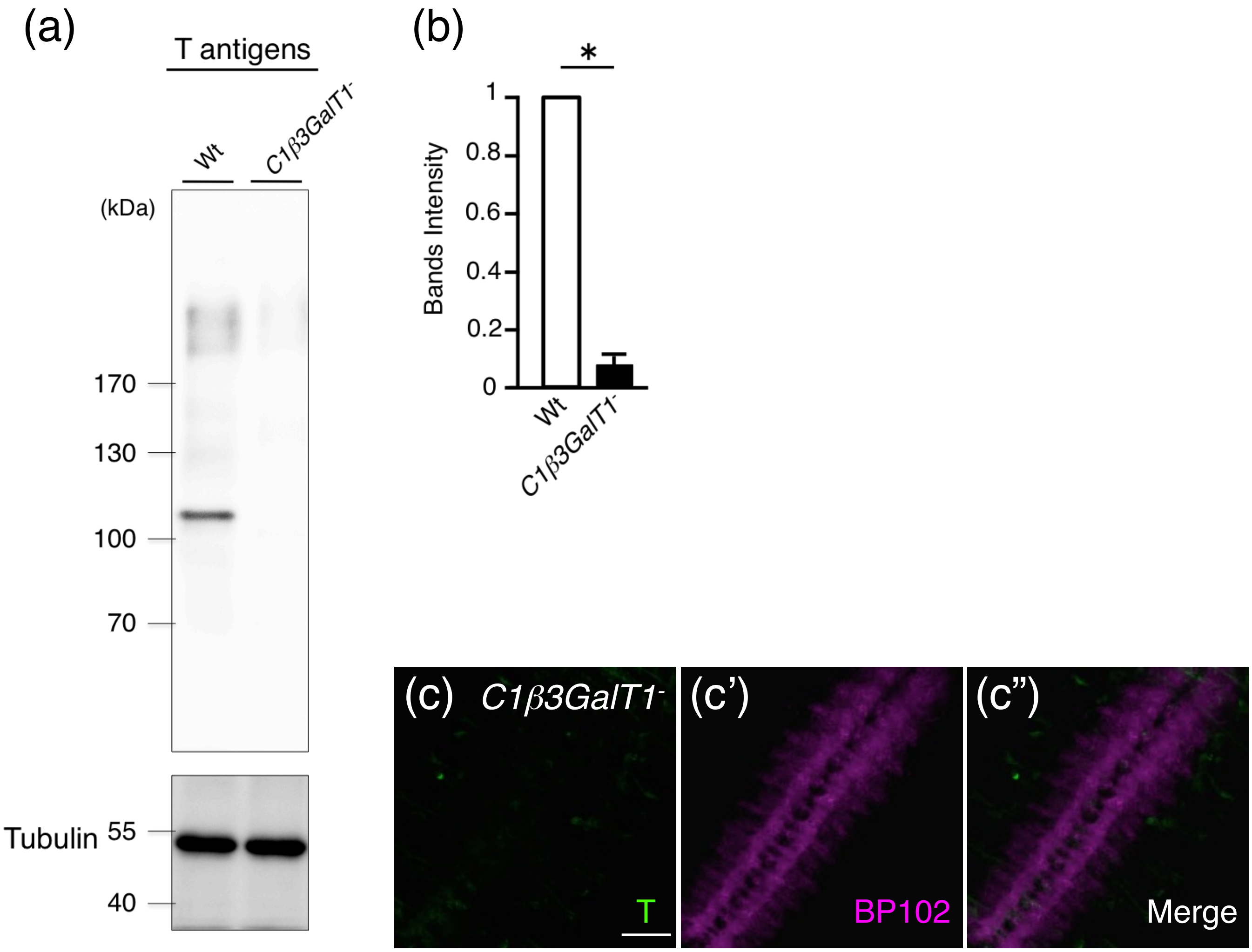

Figure S2 | T antigens are not detected in *C1β3GalT1* mutant embryos
(a) Full-length PNA lectin blots and Western blots. These blots show the amount of T antigens in extracts from wild-type and *C1β3GalT1*^{EY13370} / *C1β3GalT1*$^{2.1}$ transheterozygous mutant (*C1β3GalT1*$^{-}$) embryos at stage 16. The bands of T antigens disappeared in the *C1β3GalT1* mutant; α-tubulin was used as the internal control. (b) The mean densitometric readings of the blot analyses ± SD (n = 3, *$p = 1.1 \times 10^{-5}$) after normalization against the wild-type (value = 1). *P*-values were calculated using the Student's *t*-test. (c-c") Localization of T antigens (green) and BP102 antigens (magenta) in *C1β3GalT1*$^{-}$ mutant embryo at stage 16. T antigens were not detected in the *C1β3GalT1* mutant embryo. Scale bar: 50 μm (c).

Kinoshita *et al*. Supplementary Figure 3

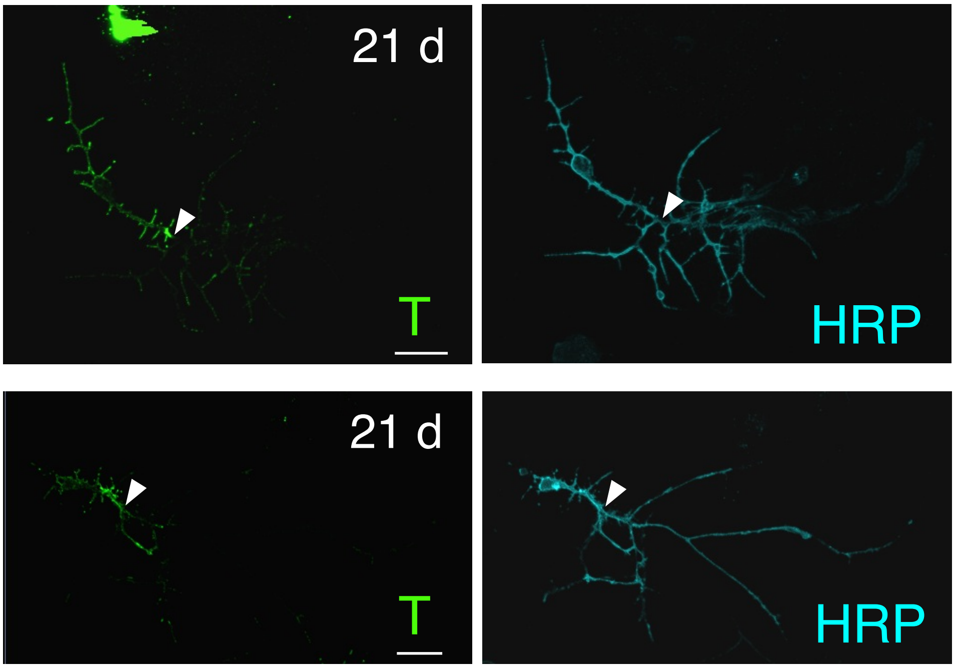

Figure S3 | Localization of T and HRP antigens on the surface of wild-type 21-d primary cultured neurons

Localization of T antigens (green) and HRP antigens (cyan) on the surface of neurons cultured for 21 d. Filled arrowheads indicate the intra-axonal boundary. Scale bar: 10 μm.

Kinoshita *et al*. Supplementary Figure 4

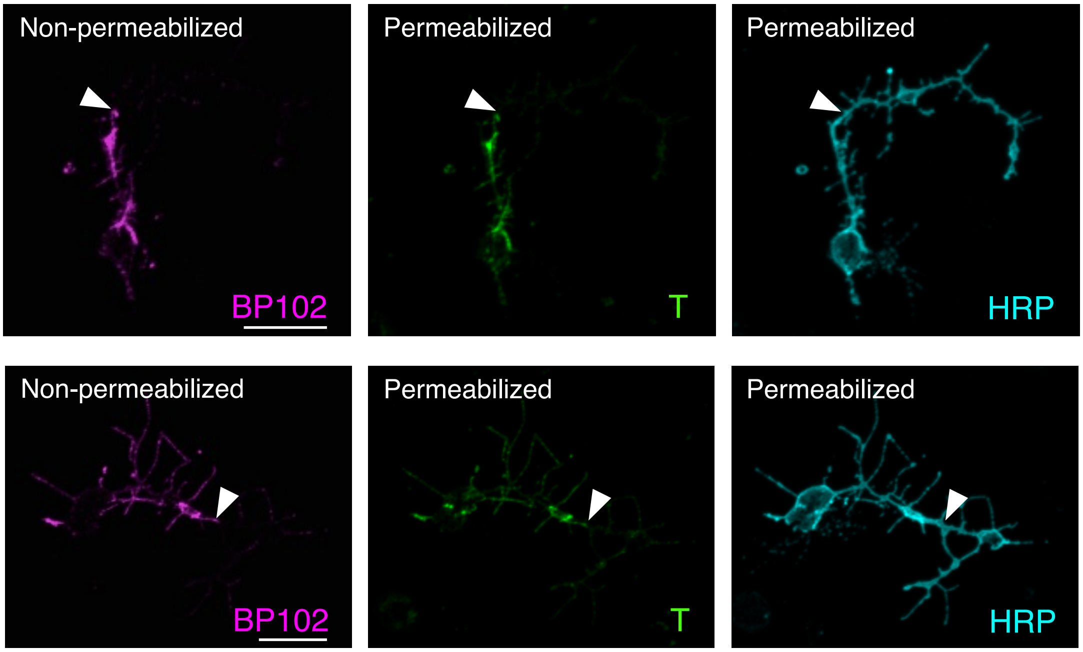

Figure S4 | The intra-axonal boundaries defined by internal T antigens and surface BP102 antigens were at the same position
Localization of surface BP102 (magenta) antigens on the cultured neurons and localization of internal T antigens (green) and HRP antigens (cyan) in the same neurons. Fig. 1b shows that the intra-axonal boundaries defined by surface T antigens and surface BP102 antigens were at the same position. In addition, the intra-axonal boundary defined by internal T antigens (green) was consistent with the boundary defined by surface BP102 antigens (magenta). These results indicate that the intra-axonal boundaries defined by surface and internal T antigens are at the same position. Filled arrowheads indicate the intra-axonal boundary. Scale bar: 10 µm.

Kinoshita *et al.* Supplementary Figure 5

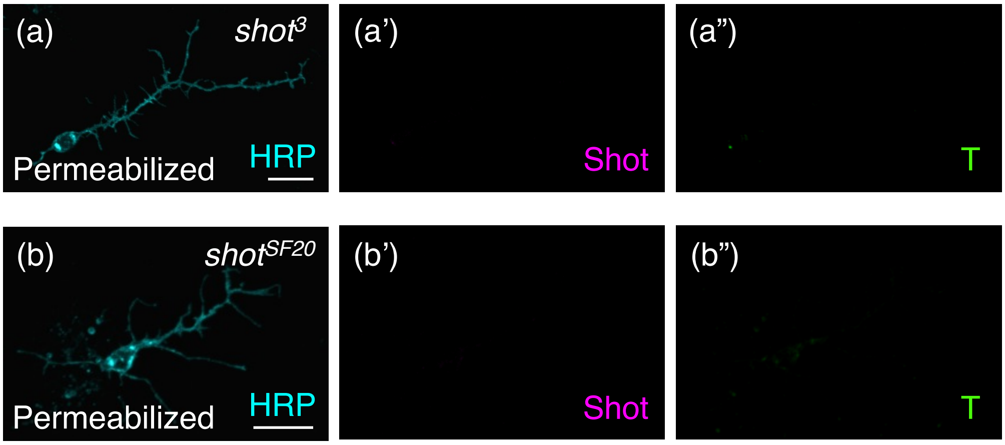

Figure S5 | In Shot mutant neurons, expressions of Shot protein was impaired

(a-b") Localization of HRP antigens (cyan), Shot (magenta), and T antigens (green) in permeabilized primary cultured neurons of *shot*^{3} (a-a") and *shot*^{SF20} (b-b") mutants. Shot was not detectable in more than 90% of both mutants neurons. HRP antigens were detectable in the same neurons, and were always localized in the whole axon. *n*= 124 and 117 for *shot*^{3} and *shot*^{SF20} mutants, respectively. Scale bar: 10 μm (a, b).

Kinoshita *et al*. Supplementary Figure 6

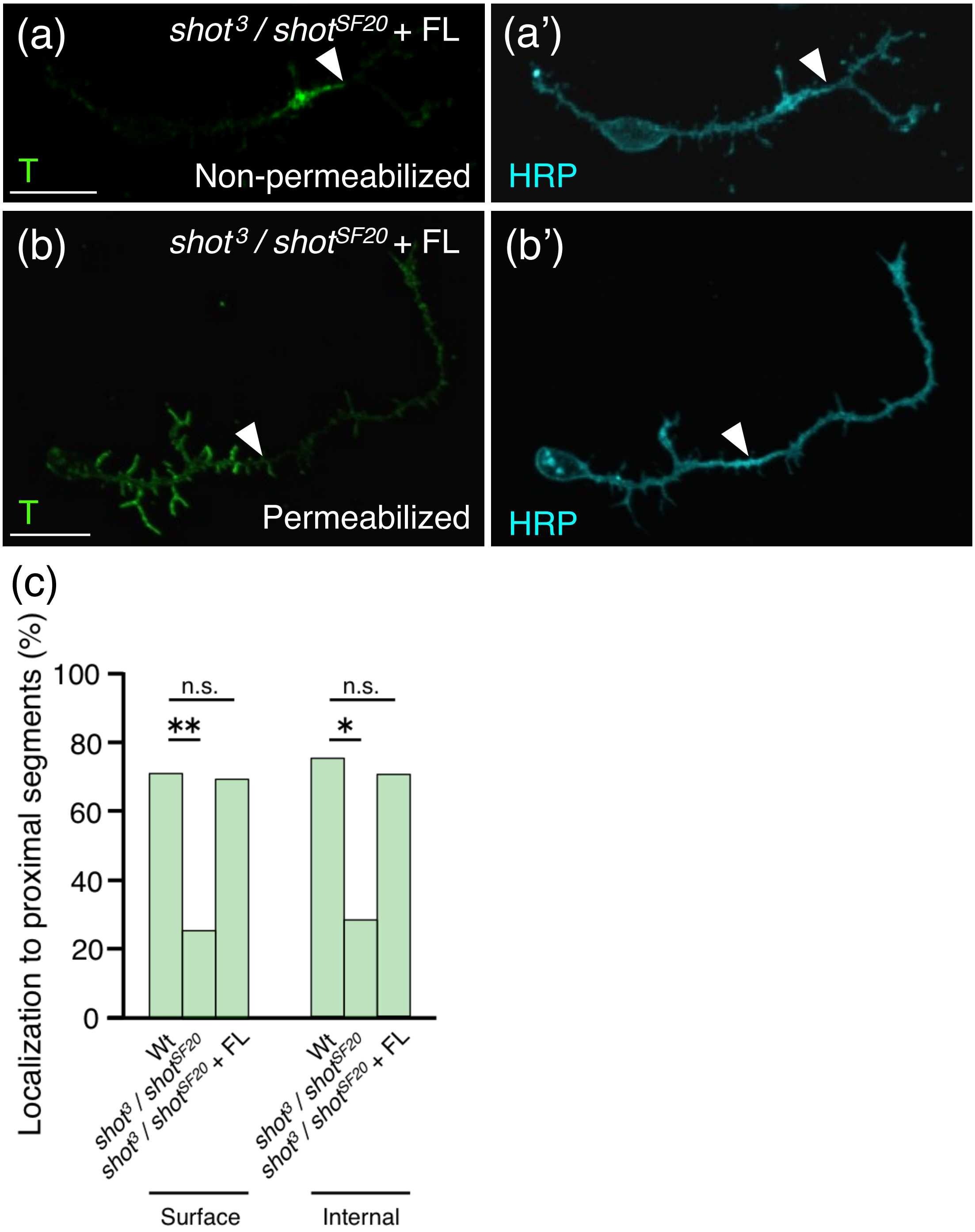

Figure S6 | Proximal localization of T antigens is rescued by neuronal expression of wild-type Shot in *shot* mutant background

(a-b') Localization of T (green) and HRP (cyan) antigens in/on *elav-Gal4* rescued neurons (*UAS-Shot-full-length; shot*^{3} / *shot*^{SF20}*; elav-Gal4* / +). T antigens localized in/on the proximal axon segments. Filled arrowheads indicate the intra-axonal boundary. (c) Histogram showing the percentage of neurons with proximal localization of T antigens in/on the wild-type, *shot*^{3} / *shot*^{SF20} mutant, and *elav-Gal4* rescued neurons. In both permeabilized and non-permeabilized *elav-Gal4* rescued neurons, proximal localization of T antigens was restored to the same level as observed in wild-type neurons. *P*-values were calculated using the G-test. $**p = 8.0 \times 10^{-14}$, $*p = 6.5 \times 10^{-10}$, n.s. > 0.4. The number of observations for each genotype are as follows: (surface) wild-type, $n = 120$; *shot*^{3} / *shot*^{SF20}, $n = 152$; *elav-Gal4* rescued, $n = 96$; (internal) wild-type, $n = 93$; *shot*^{3} / *shot*^{SF20}, $n = 91$; *elav-Gal4* rescued, $n = 105$. Scale bar: 10 μm (a, b).

Kinoshita *et al*. Supplementary Figure 7

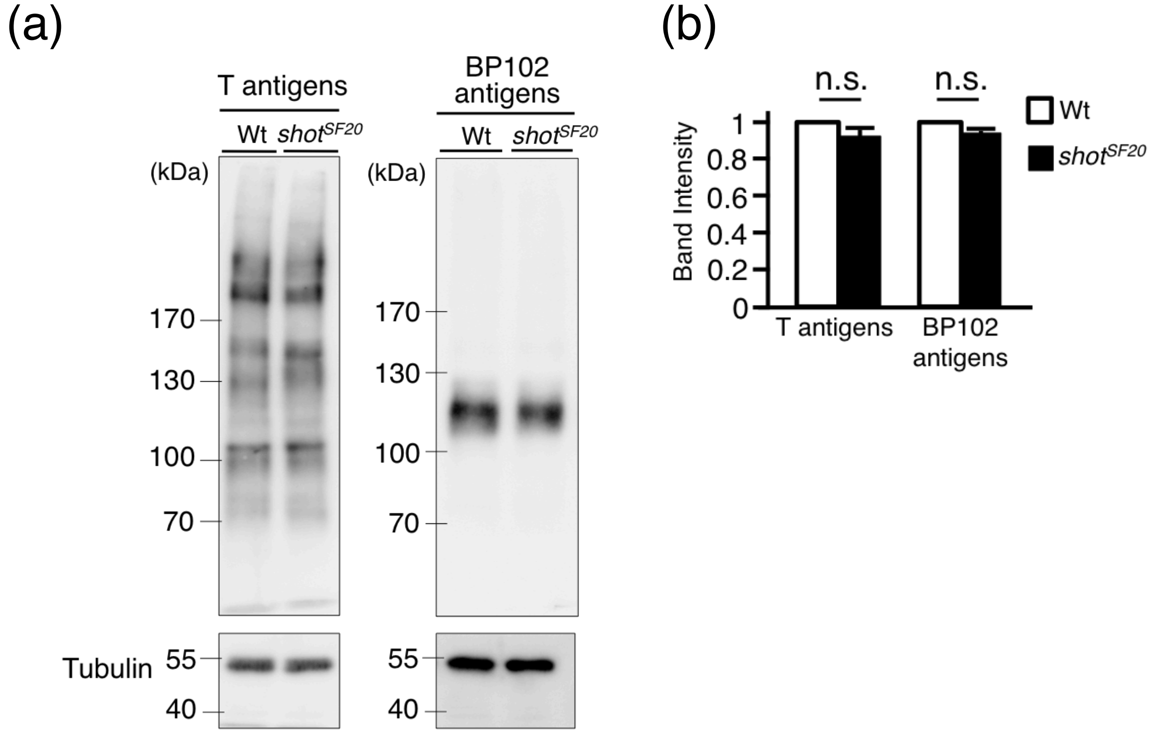

Figure S7 | PNA lectin and Western blot analyses of the total amount of T antigens and BP102 antigens in wild-type and *shot^{SF20}* mutant embryos
(a) Full-length PNA lectin blots and Western blots with mouse BP102 anti-CNS axons antibody. These blots show the amount of T antigens and BP102 antigens in extracts from wild-type and *shot^{SF20}* mutant embryos at stage 16. The amounts of T antigens and BP102 antigens were the same for the wild-type and the *shot^{SF20}* mutant; α-tubulin was used as the internal control. (b) Histogram showing mean densitometric readings of the blot analyses ± SD (n = 3, n.s. > 0.1) after normalization against the wild-type (value = 1). *P*-values were calculated using the Student's *t*-test.

Kinoshita *et al*. Supplementary Figure 8

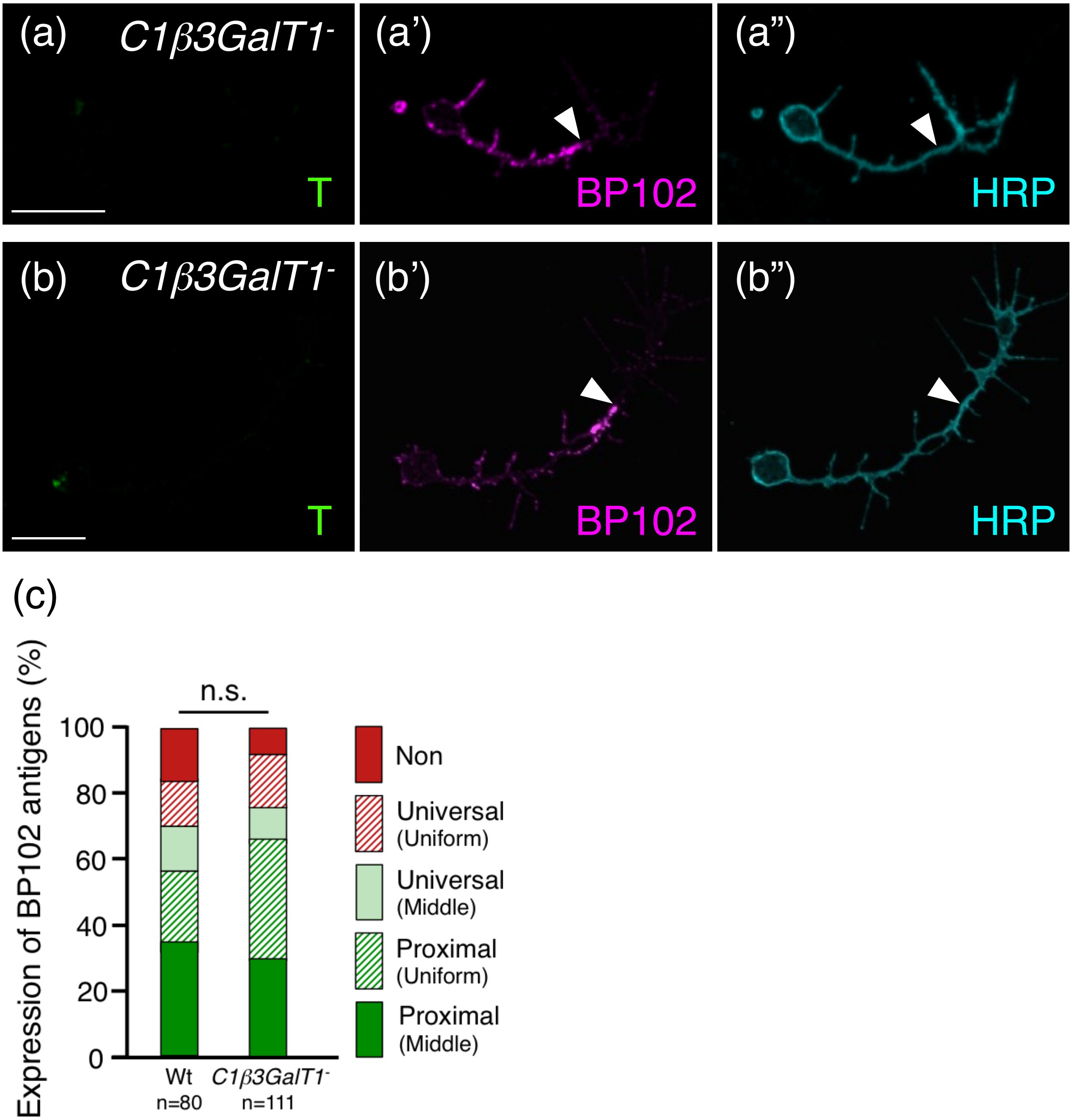

Figure S8 | Proximal localization of BP102 antigens is not disturbed in *C1β3GalT1⁻* mutant neurons

(a-b") Localization of T (green), BP102 (magenta), and HRP (cyan) antigens on the surface of *C1β3GalT1*^{EY13370} / *C1β3GalT1*$^{2.1}$ transheterozygous mutant (*C1β3GalT1*$^{-}$) neurons. BP102 antigens localized on the surface of proximal axon segments. Filled arrowheads indicate the intra-axonal boundary. (c) Histogram showing the localization of BP102 antigens on the *C1β3GalT1*$^{-}$ mutant neurons. The proximal localization of BP102 antigens was not changed significantly in the *C1β3GalT1*$^{-}$ mutant neurons. *P*-values were calculated using the G-test. n.s. = 0.12. Scale bar: 10 μm (a, b).

Supplementary References

1. Lin, Y. R., Reddy, B. V. V. & Irvine, K. D. Requirement for a core 1 galactosyltransferase in the *Drosophila* nervous system. *Dev. Dyn.* **237,** 3703–3714 (2008).
